# Directed Migration of Positively Selected Thymocytes Visualized in Real Time

**DOI:** 10.1371/journal.pbio.0030160

**Published:** 2005-05-03

**Authors:** Colleen M Witt, Subhadip Raychaudhuri, Brian Schaefer, Arup K Chakraborty, Ellen A Robey

**Affiliations:** **1**Division of Immunology, Department of Molecular and Cell Biology, University of CaliforniaBerkeley, CaliforniaUnited States of America; **2**Department of Chemical Engineering, University of CaliforniaBerkeley, CaliforniaUnited States of America; **3**Department of Microbiology and Immunology, Uniformed Services University of the Health SciencesBethesda, MarylandUnited States of America; University of MinnesotaUnited States of America

## Abstract

Development of many vertebrate tissues involves long-range cell migrations. In most cases, these migrations have been inferred from analysis of single time points and the migration process has not been directly observed and quantitated in real time. In the mammalian adult thymus, immature CD4^+^CD8^+^ double-positive (DP) thymocytes are found in the outer cortex, whereas after T cell antigen receptor (TCR) repertoire selection, CD4^+^CD8^–^ and CD4^–^CD8^+^ single-positive (SP) thymocytes are found in the central medulla. Here we have used two-photon laser-scanning microscopy and quantitative analysis of four-dimensional cell migration data to investigate the movement of thymocytes through the cortex in real time within intact thymic lobes. We show that prior to positive selection, cortical thymocytes exhibit random walk migration. In contrast, positive selection is correlated with the appearance of a thymocyte population displaying rapid, directed migration toward the medulla. These studies provide our first glimpse into the dynamics of developmentally programmed, long-range cell migration in the mammalian thymus.

## Abstract 2

Two-photon laser-scanning microscopy reveals the change from random motion to directed migration that occurs when thymocytes undergo positive selection.

## Introduction

Although it is known that thymocytes relocalize from the cortex to the medulla after positive selection, the means by which this relocalization occurs is largely unknown [1–3]. For example, thymocytes may migrate by random walk throughout the cortex, with selected thymocytes being captured at the medulla by short-range guidance cues. Alternatively, thymocytes may migrate directionally across the cortex in response to long-range cues emanating from the medulla. Directional migration in response to such long-range cues may be induced by positive selection or may be a property of all cortical thymocytes. To address these and other questions, we undertook a real-time analysis of thymocyte migration in intact thymic lobes.

## Results/Discussion

**Figure 1:**
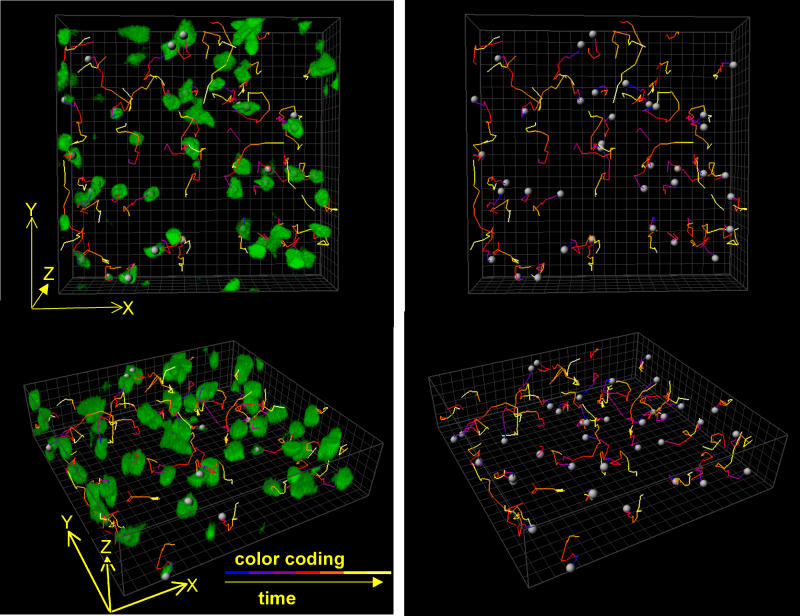
Tracking Thymocyte Migration in 3D Tracking software identifies the positions of individual thymocytes over time. Trajectories of individual cells are shown as tracks, which are color coded to indicate increasing time from blue (start of imaging) to yellow (end of imaging) (see Videos S1–S4). Left panels show fluorescent signal from thymocytes at a single time point superimposed on cell tracks. Right panels show the positions of thymocytes (indicated by spheres) at a single time point. Top panels show a projection in which the *z*-axis is perpendicular to the viewer. In the bottom panels the image is rotated to display the *z* dimension.

In order to track migrating thymocytes in situ, we generated chimeric mice in which a fraction of thymocytes express green fluorescent protein (GFP). We devised a protocol, based on a previously described method [4], in which adult bone marrow from GFP transgenic mice [5] is injected into newborn mice to generate partial GFP hematopoietic chimeras without the use of irradiation (see Materials and Methods). The GFP^+^ thymocytes generated in this way comprised approximately 1% of total thymocytes and showed expected developmental profiles by flow cytometric analysis (Figure S1). At 4.5 to 5.5 wk of age, chimeric thymic lobes were harvested and imaged while being perfused with warmed oxygen-supplemented media. Imaging of intact lymph nodes under similar conditions revealed identical T cell and dendritic cell motility and behavior to that observed from intravital imaging of lymph nodes [6–9]. The objective was positioned above the center of the thymic lobe such that the movement of the stage (*z* direction) was perpendicular to the thymic capsule (Figures S2 and S3). Regions of thymic cortex 104 × 104 × 40 μm were scanned at tissues depths between 80–200 μm below the surface of the capsule. The scan sequence was repeated every 37 s for up to 33 min to generate four-dimensional (4D) datasets (*x, y, z,* and time) (Videos S1 and S2). The datasets were analyzed with a combination of 4D cell-tracking software (Figure 1; Video S3) and mathematical treatment of the cell tracks.

**Figure 2:**
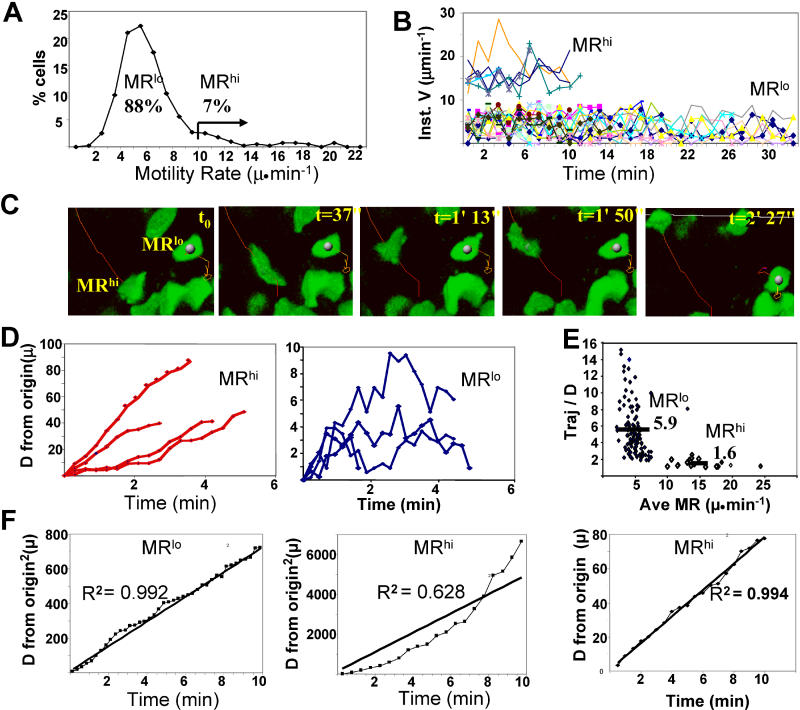
Two Distinct Migratory Behaviors within Wild-Type Cortical Thymocytes (A) Histogram showing the frequency distribution of average motility rates (MR) for cortical thymocytes compiled from over 1,250 tracked cells from four independently imaged thymic lobes. The vast majority of cells exhibited speeds ranging from 3 to 8 μm/min (MR^lo^). Approximately 7% exhibited speeds of 10 μm/min or greater (MR^hi^). Cells migrating between 10–13 μm/min represented approximately 5% of cortical thymocytes, and those with speeds of 14 μm/min or greater represented approximately 2% of cortical thymocytes. (B) Instantaneous velocities versus time for representative MR^hi^ and MR^lo^ cells. Data are representative of 53 MR^hi^ cells and more than 200 MR^lo^ cells analyzed. No conversions between MR^hi^ or MR^lo^ behaviors were observed over a combined imaging time of more than 30 h. (C) Five successive time frames showing the morphology associated with propulsion for an MR^hi^ and an MR^lo^ cell (Video S5). (D) Graph of displacement versus time for four individual MR^hi^ and MR^lo^ cells. (E) Graph of directional index (Traj/D) versus average motility rate. The bars indicate the average values for Traj/D computed from 50 MR^hi^ and 466 MR^lo^ cells**.** (F) Graph of MR^lo^ cells (left), but not MR^hi^ cells (middle), shows linear relationship between the square of the displacement from origin versus time, indicative of random walk. Right graph shows a linear relationship between displacements from origin (as opposed to their square) with increasing time for MR^hi^ cells, indicative of ballistic motion (right). Analysis was done on 466 MR^lo^ cells and 50 MR^hi^ cells from three independently imaged thymic lobes.

Analysis of the motility rates of individual GFP cortical thymocytes (Figure 2A) showed that the vast majority of cells (88% of total cells) moved at low motility rates between 3–8 μm/min (MR^lo^) and exhibited peak instantaneous velocities of up to 9 μm/min (Figure 2B). A small subset of imaged cells (approximately 7%) moved at motility rates that were 10 μm/min or greater (MR^hi^) and exhibited peak instantaneous velocities of up to 28 μm/min (Figure 2A and 2B; Video S4). The distribution of motility rates among cortical thymocytes was highly reproducible between samples (Figure S4). The instantaneous velocities for each group showed no interconversion of motility rates throughout imaging (Figure 2B), suggesting that these differing motility behaviors represented distinct cell populations.

Further analysis showed striking differences between MR^lo^ and MR^hi^ cells with regard to their morphology and with other aspects of their migratory behavior. For example, MR^hi^ cells displayed a highly polarized morphology with a well-defined leading edge and uropod, and moved by a series of lurches followed by contraction (Figure 2C; Video S5). In contrast, MR^lo^ cells exhibited a more spherical morphology that lacked obvious polarization. Propulsion by MR^lo^ cells involved only a modest protrusion of the cell's leading edge. These properties exclusively segregated with motility rates and remained constant over the entire imaging time. Also, whereas MR^lo^ cells showed frequent pausing during the course of their trajectories, MR^hi^ cells were never observed to pause (see Video S3; data not shown).

Examination of a cell's displacement from origin relative to time can provide additional insight into the migratory behavior of cells. Individual MR^hi^ cells exhibit a linear relationship between displacement and time. In contrast, the displacement from origin for MR^lo^ cells revealed numerous turns back toward cell origin (Figure 2D). The turning behavior of thymocytes is of interest in part because it may reflect the interaction of thymocytes with other cells or structures in the tissue environment. For example, thymocytes engaged in dynamic contacts with MHC-bearing stromal cells during positive selection and T cells contacting antigen-bearing dendritic cells in intact lymph nodes turn frequently and show little displacement over time [7–10]. We quantitated the degree of turning by computing the total length of a cell's trajectory divided by the absolute value of its displacement from origin. If a cell's migratory path shows little to no deviation from a straight line, this ratio will be close to one. An analysis of this directional index for each population showed a ratio of 5.9 for MR^lo^ cells as compared to 1.6 for MR^hi^ cells (Figure 2E). Taken together with the frequent pausing observed for MR^lo^ cells, but not for MR^hi^ cells, these results suggest that the MR^lo^ thymocytes interact with their environment more extensively than do MR^hi^ thymocytes.

A major aim of this study was to determine whether the localization of mature thymocytes to the medulla involves directed inward migration across the cortex, and if so, whether directed migration is a property of all cortical thymocytes or only thymocytes that have been selected to mature. To examine this question, we used graphical techniques borrowed from diffusion mechanics to distinguish movement by random walk versus directed migration [11] (see Materials and Methods). Analysis of 1,250 MR^lo^ cells showed that the relationship between displacement from origin with respect to time was consistent with random walk statistics (Figure 2F). In contrast, a graph of the mean displacement from origin (as opposed to the square of the displacement) versus time for MR^hi^ cells showed a linear relationship, indicative of directed migration. Thus, MR^lo^ and MR^hi^ thymocytes use distinct modes of migration as they move through the cortex.

**Figure 3:**
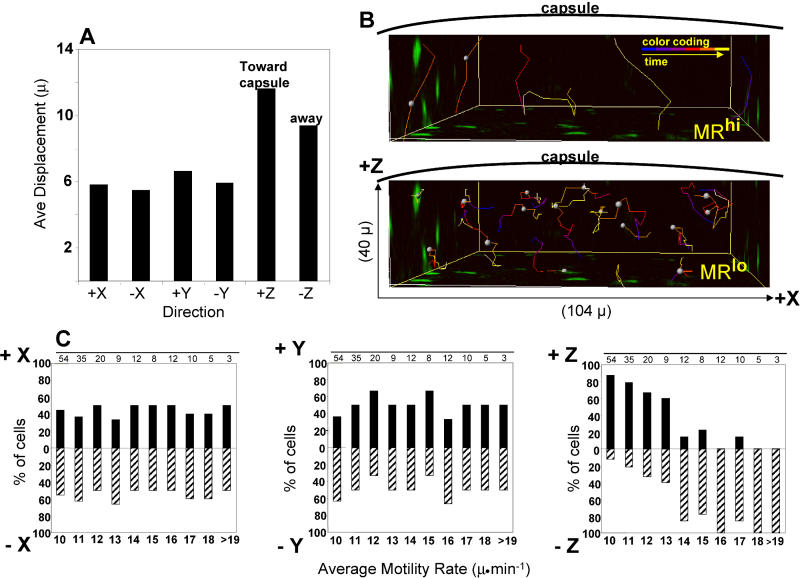
MR^hi^ Thymocytes Show Preferential Movement Perpendicular to the Thymic Capsule (A) Bar graph showing the average displacement in each direction by wild-type MR^hi^ cells in a 3-min interval. Data shown were computed from 53 MR^hi^ cells from four independently imaged thymic lobes. Data from individual runs are shown in Figure S3. (B) The upper image is rotated to display the *x* and *z* dimensions showing tracks of MR^hi^ cells. Five of six MR^hi^ tracks show preferential orientation in the *z* direction. The lower image shows tracks of MR^lo^ cells from same dataset. (C) The results of step analysis (see Materials and Methods) on 172 MR^hi^ cells. Thymocytes are grouped according to their average motility rate (displayed on *x*-axis) and percentage of cells moving in either the positive or negative direction is displayed on the *y*-axis. Data are compiled from four independently imaged thymic lobes.

The observation that MR^hi^ cells moved by directed migration is consistent with the possibility that these cells are being directed to migrate toward the medulla. If this were the case, we would expect their trajectories to show a common orientation in the –*z* direction (away from the capsule). To examine this question, we used two methods of statistical analysis (see Materials and Methods). First, vector analysis was performed in which the average displacement per cell in each direction of three-dimensional (3D) space for a fixed time interval of 3 min was calculated (Figure 3A). If MR^hi^ cell tracks were randomly oriented in the cortex, then the average displacement in each of the six directions (+*x,* –*x,* +*y,* –*y,* +*z,* and –*z*) would be similar. On the other hand, if there were common directionality for MR^hi^ cell trajectories, we should see an increase in average displacement values for the preferred direction. As shown in Figure 3A (and Figure S5), although the average displacements in the *x* and *y* directions were similar, there was greater displacement in both +*z* and -*z* directions with the greatest bias in the +*z* direction (toward the capsule). Figure 3B provides a visual representation of track orientation shown along the *x* and *z* directions for both MR^lo^ (top panel) and MR^hi^ cells (lower panel).

To confirm and extend these results, we performed a step analysis on MR^hi^ cells (see Materials and Methods) in which individual thymocytes were scored as showing net displacement in the positive or negative direction along each of three axes (*x, y,* and *z*). This analysis allowed us to correlate the tendency of thymocytes to migrate in a particular direction with their motility rates (Figure 3C). As expected, thymocytes showed equal tendency to displace in the positive and negative directions along the *x* and *y* axes. In striking contrast, cells with motility rates greater than 13 μm/min were consistently scored as moving in the –*z* direction (away from the capsule, toward the medulla). Interestingly, the vast majority of cells with motility rates in the range of 10–12 μm/min were scored as moving in the +*z* direction (toward the capsule). We suspect that the population with intermediate motility rates is comprised of CD4^–^CD8^–^ double-negative (DN) thymocytes based on published evidence for outward migration of DN thymocytes in the adult thymus [12,13].

CD4^+^CD8^+^ double-positive (DP) thymocytes express clonally variable versions of the T cell antigen receptor (TCR). Following somatic V(D)J rearrangement and cell surface expression of the αβTCR, cortical thymocytes test out their antigen receptors for their ability to bind self-peptide and MHC proteins expressed in the thymus. A small fraction of thymocytes expressing TCR with moderate avidity for self-peptide MHC receive signals that allow them to differentiate into more mature medullary CD4^+^CD8^–^ or CD4^–^CD8^+^ thymocytes, a process known as positive selection [2,3,14]. The low frequency of cortical thymocytes with motility rates greater than 13 μm/min, together with their biased movement away from the capsule, led us to hypothesize that these cells might represent thymocytes that had successfully undergone positive selection.

**Figure 4:**
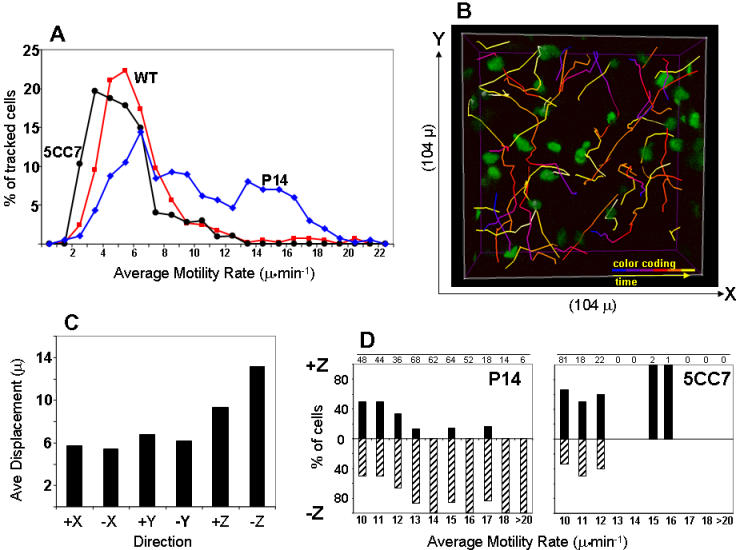
Positive Selection Leads to an Increased Frequency of MR^hi^ Thymocytes Migrating away from the Thymic Capsule (A) A histogram showing the frequency distribution of average motility rates for positively selecting (blue, P14) and nonselecting (black, 5CC7) transgenic thymocytes compiled from over 1,200 P14 and 875 5CC7 thymocytes from, respectively, four and three independently imaged thymic lobes. Data (from Figure 2A) from wild-type (WT) thymocytes (red) were overlaid for comparison. P14 cells moving at motility rates greater than 13 μm/min were 34% of total imaged thymocytes (Videos S6 and S7) compared to approximately 1% of wild-type cortical thymocytes. Analysis of 5CC7 thymocytes showed nearly complete absence of cells moving at motility rates greater than 13 μm/min, a value that differed significantly (*p* = 0.002) from wild-type cortical thymocytes. (Video S8) (B) Image showing trajectories of representative P14 MR^hi^ cells. Note tracks for P14 thymocytes are relatively linear compared to the tracks of wild-type thymocytes (see Figure 1). (C) Bar graph showing the average displacement per cell moved in each direction over a 3-min time interval (left). Data was computed from more than 100 P14 MR^hi^ cortical thymocytes compiled from four independent experiments. Data from individual runs are shown in Figure S5. (D) Results of step analysis on 412 P14 thymocytes as a function of motility rate are shown (left). Results of step analysis on 123 5CC7 thymocytes are shown for comparison (right). P14 cells moving at MR greater than 13 μm/min showed strong bias for movement in the –*z* direction (away from capsule) whereas 5CC7 thymocytes showed random use of both +*z* and –*z* directions.

To test this hypothesis, we generated chimeric mice in which a small fraction (approximately 1%) of thymocytes expressed both GFP and rearranged TCR transgenes that do or do not allow positive selection. As a positive-selecting TCR, we used the class I MHC-restricted P14 TCR transgene, which promotes the development of mature CD8 T cells in the H2^b^ (B6) background [15]. As a nonselecting TCR, we used the 5CC7 TCR transgene, which leads to neither positive nor negative selection in the B6 background [16]. Analysis of the motility rate distribution of cortical thymocytes expressing transgenic TCRs showed a striking correlation between positive selection and the frequency of thymocytes with high motility rates (Figure 4A and Figure S6). A total of 34% of cortical thymocytes expressing the P14 TCR had motility rates greater than 13 μm/min compared to approximately 2% for wild-type thymocytes expressing diverse TCRs. In addition, thymocytes expressing the nonselecting 5CC7 TCR showed a nearly complete absence of cortical thymocytes moving at speeds greater than 13 μm/min. In fact, only three of the 1,275 5CC7 thymocytes that were analyzed exhibited motility rates greater than 13 μm/min. These values are significantly different from wild-type cortical thymocytes in which 50 of 1,670 thymocytes exhibited motility rates greater than 13 μm/min (*p* = 0.002). Moreover, although the majority of rapidly migrating wild-type thymocytes were migrating away from the capsule (44 of 50), all three of the rapidly migrating 5CC7 thymocytes were moving toward the capsule (Figure 4).

Our studies show that positive selection leads to a rapid directional migration pattern and are consistent with earlier studies showing that activated CD4^+^CD8^+^ cells migrate rapidly in vitro [17]. In an earlier study of thymocyte–stromal cell interactions in reaggregate thymic organ culture, we did not note a major difference in overall motility rates between positively selected and wild-type thymocytes [10]. This may be due to the fact that reaggregate thymic organ cultures lack the normal spatial distribution of chemokines and other guidance cues that are likely to control thymocyte migration patterns.

As in the case of MR^hi^ cells of wild-type mice, P14 MR^hi^ cells displayed a highly polarized morphology, and their trajectories showed very little turning, with no incidence of pausing (Figure 4B and data not shown). In addition, P14 MR^hi^ thymocytes displayed greater displacement in the positive and negative *z* directions (see Figure 4C), and step analysis showed that the vast majority of cells moving at rates greater than 13 μm/min moved away from the capsule (Figure 4D). The motility rates and directionality of P14 thymocytes were highly reproducible between samples (Figures S6 and S7).

When considering thymocytes with intermediate motility rates (10–12 μm/min), there were two notable differences between P14 and wild-type thymocytes (Figure 4A and 4D). First, the frequency of these intermediate motility thymocytes was higher among P14 compared to wild-type cortical thymocytes (Figure 4A). In addition, whereas the majority of wild-type thymocytes of intermediate motility migrated toward the capsule (see Figure 3C, right panel), this trend was less clear among P14 thymocytes (Figure 4D, left panel). These differences could be explained by the proposition that thymocytes with intermediate motility rates consist of a mixture of outwardly migrating CD4^–^CD8^–^ DN thymocytes and inwardly migrating positively selected CD4^+^CD8^+^ thymocytes, with the DN subset predominating in wild-type samples. In P14 samples, the increase in the number of intermediate motility, inwardly migrating thymocytes could be attributed to an increase in the fraction of thymocytes undergoing positive selection.

Importantly, we observed directional migration of MR^hi^ thymocytes in each dataset corresponding to a region of the cortex that extends from, approximately, 80 μm to 200 μm below the thymic capsule. This suggests that thymocyte migration is directed by guidance cues that extend over a large area of the cortex. Although the nature of these guidance cues is currently unknown, there are a number of chemokines expressed in the medulla whose corresponding receptors are upregulated during positive selection [18–23]. These include CCL19/CCL21, whose receptor, CCR7, is upregulated on activated CD4^+^CD8^+^ thymocytes [19,24]. Moreover, gain and loss of function mutations have implicated CCR7 in the positioning of mature SP thymocytes to the medulla [24,25]. The contribution of CCR7 and other chemokine receptors in controlling the thymocyte migration patterns described here will be an important area for future investigation.

A cortical thymocyte must travel a distance of hundreds of microns in order to reach the medulla. Based on the average distance from the capsule to the medulla in the adult mouse thymus, and the speed and directionality reported here, we estimate that a typical MR^hi^ thymocyte that we image in the cortex could arrive at the medulla in 1 to 2 h. This short time period for migration to the medulla is in contrast to the estimates of 2–3 d for a CD4^+^CD8^+^ thymocyte to complete positive selection [26,27]. Moreover, we have previously shown that thymocytes frequently pause and turn during MHC-driven contacts with stromal cells [10], behaviors that differ strikingly from the behavior of MR^hi^ thymocytes described here. Based on these considerations, we suspect that thymocytes moving at rates greater than 13 μm/min represent cells at a relatively late stage in the positive-selection process and that migration from the cortex to the medulla may not require continuing MHC engagement. In contrast, the MR^lo^ cells are likely to include thymocytes that are actively engaging thymic stromal cells and receiving MHC-driven TCR signals. Future analysis of the signaling events and migratory patterns of these slowly migrating thymocytes may provide further insights into the process of positive selection in the thymus.

## Materials and Methods

### 

#### Generation of GFP chimeric mice

Mice expressing a GFP transgene driven by the ubiquitin promoter [5] were used as bone marrow donors for the generation of GFP hematopoietic stem cell chimeras using a modification of a previously described procedure [4]. Whole bone marrow from a single adult GFP^+^ mouse was aseptically harvested and resuspended into a single cell suspension in sterile Hanks' Balanced Salt Solution (Mediatech Cellgro). A total of 2–3 × 10^6^ bone marrow cells were injected into newborn mice (C57Bl/6) in a volume of 70 μl. The first injection was done at 12–24 h after birth and repeated every 2–3 d for a total of four injections. Resulting chimeric mice expressed GFP in 1–2% of their thymocytes. P14 TCR transgenic mice [15] on a Rag2^–/–^B6 background were obtained from Taconic. P14^+/+^Rag2^–/–^ mice were crossed with UBI-GFP transgenic mice to generate P14^+/-^Rag2^+/–^GFP^+/–^ mice, and these mice were then intercrossed to generate P14^+/+ or +/–^Rag2^–/–^GFP^+/+ or +/–^ mice. 5CC7 TCR transgenic mice [16] on a Rag2^–/–^B10 background were obtained from Taconic and were crossed once with UBI-GFP transgenic mice to generate 5CC7^+/–^GFP^+/–^Rag2^+/–^mice. Bone marrow from adult double transgenic mice was used to generate chimeric mice as described above.

#### Two-photon imaging of intact thymic lobes

Thymi from 4.5–5.5 wk-old GFP chimeric mice were quickly harvested, lobes were separated, and the dorsal face of the lobe was adhered to 22 × 22 mm cover glass with single drop of Vetbond tissue adhesive (see Figure S2). Cover slip with lobe was immediately placed into a 60 × 15 mm polystyrene Petri dish containing Dulbecco's modified Eagle's medium (DMEM) without phenol red (Mediatech Cellgro). Petri dish was placed into a heated ring, and the sample was perfused with warmed media bubbled with a blend of 95% O_2_ and 5% CO_2_. Sample was maintained under perfusion and held at 36.5 °C to 37.5 °C throughout imaging. Thymic lobes maintained under these conditions for up to 6 h showed no changes in cell motility and no indications of tissue deterioration. Imaging was performed as previously described [8] using an upright Zeiss NLO 510 microscope equipped with a MaiTai Ti:Sapphire laser (Spectra-Physics). For each dataset, 20–33 min of imaging was performed with the objective oriented over the top center of the thymic lobe (corresponding to the ventral side of the organ; see Figures S2 and S3). A total of 20 optical slices were acquired at 2-μm step intervals with a total acquisition time of 36.7 s/*z* stack. Using Imaris Bitplane software, *z* stacks (104 × 104 × 40 μm in dimension) were processed into 3D images and reiterated through time to generate a 3D movie of thymocyte migration. In most experiments, data were acquired as “blocks” of stacked movies; a first movie was made at a maximum depth below the capsule with a second movie acquired immediately above the bottom movie (–180 to –80 μm; see Figure S3B). Analysis of HNE-stained thymic tissue sections indicated that the area imaged invariably corresponded to cortex (data not shown).

#### 4D data analysis

The 4D cell tracking was performed on 75–300 cells per movie using Imaris Bitplane software which identifies the *x, y,* and *z* coordinates for each cell at each given time point. These statistics were exported into an Excel spreadsheet for analysis. Average motility rates (MR) were computed as the total length of migratory path divided by the total time of tracking. The extent to which a cell's migratory path deviated from a straight line was quantitated as the total length of a cell's trajectory divided by the total displacement from origin. For the determination of movement by random walk for MR^lo^ cells, the mean-square displacement from origin was shown to be proportional to time. This relationship is given by 〈r^2^〉 = 6Dt, where r = the displacement from origin, 〈 〉 denotes the average r over numerous events at time t, and 6D is the motility coefficient [9], which characterizes the spread of cells in three dimensions. Movement by directed migration for MR^hi^ cells was indicated by a linear relationship between mean displacement from origin (as opposed to its square) and time when computed over many events. The correlation coefficient *R^2^* for the best-fit line was computed in Excel using the least squares method. *R^2^* at *p* = 0.001 is statistically significant at values of 0.801 or greater. For displacement analyses of MR^hi^ cells, the average displacement per cell in each direction was calculated from 3 min of tracking. For step analyses, thymocytes were grouped according to their average motility rates and scored as showing net displacement in the positive or negative directions along each of three axes (*x, y,* and *z*). The percentage of cells in each motility rate category which moved in the positive and negative directions was then graphed as a function of average motility rate. The computation of statistical significance between the frequencies of 5CC7 TCR thymocytes with high motility rates as compared to wild-type thymocytes was done in Excel using a paired *t* test.

## Supporting Information

Figure S1 Developmental Profiles of GFP+ Thymocytes from Chimeric Mice Representative profiles obtained by flow cytometric analysis of P14 and 5CC7 chimeric thymii. As expected, GFP^+^-gated P14 thymocytes (top row) showed high levels of TCR within the CD4^+^CD8^+^ population and a high percentage of CD8^+^ SP thymocytes, indicating a high frequency of positive selection. As expected for expression of 5CC7 in a nonselecting host (bottom row), thymocytes remained arrested at the CD4^+^CD8^+^ stage of development and fail to upregulate TCR.
(128 KB TIF).

Figure S2 Orientation of Imaging Relative to Thymic Lobes In Vivo Thymic lobes are depicted in their normal position relative to the heart. Thymic lobes were surgically removed and separated, and then the dorsal side (side facing the heart) of thymic lobe was adhered to glass cover slip. Imaging (see Figure S3A and S3B) was performed with the objective positioned over the center of the ventral side of lobe.
(223 KB TIF).

Figure S3 Two-Photon Imaging of Thymocyte Migration in Intact Thymic Lobes (A) Explanted GFP chimeric thymic lobe was placed in oxygen-perfused media and maintained at 37 °C throughout experiment. Objective was placed directly over the top of lobe and a total of 20 optical slices at 2-μm step intervals were acquired, which generated *z* stacks of 104 x 104 x 40 μm in the *x, y,* and *z* directions. The *z* stack acquisition was repeated every 37 s for 20–33 min. Stacks were rendered into 3D images and processed through time to yield 4D datasets (see Videos S1–S8).
(B) In most cases, a stack of movies was generated to increase the effective area of imaging. A bottom movie was generated by imaging starting at –160 to –200 μm below capsule and then a second movie was generated starting 2 μm above the bottom movie.
(334 KB TIF).

Figure S4 Frequency Distribution of Average Motility Rates for Wild-Type Cortical Thymocytes Histograms showing the frequency distribution of average motility rates for wild-type cortical thymocytes were obtained from four individual runs. Compiled data are shown in Figure 2A.
(76 KB TIF).

Figure S5 Displacement Analyses of Wild-Type MR^hi^ Cells Results of displacement analyses of wild-type MR^hi^ cells from 4 individual experiments are shown. Bar graphs show the average displacement per MR^hi^ cell moved in each direction in a 3-min interval. Data shown were computed from 11–16 MR^hi^ cells from each dataset. The four runs made up two separate stacks of movies (see Materials and Methods and Figure S3B). Compiled data are shown in Figure 3A.
(73 KB TIF).

Figure S6 Frequency Distribution of Average Motility Rates for P14 Cortical Thymocytes Histograms showing the frequency distribution of average motility rates for P14 cortical thymocytes were obtained from four individual runs. Compiled data are shown in Figure 4A.
(79 KB TIF).

Figure S7 Displacement Analyses of P14 MR^hi^ Cells Results of displacement analyses of P14 MR^hi^ cells from four individual experiments are shown. Bar graphs show the average displacement per MR^hi^ cell in each direction in a 3-min interval. Data shown were computed from 29–35 MR^hi^ cells from each dataset. Compiled data are shown in Figure 4C.
(76 KB TIF).

Video S1 GFP Thymocytes within an Intact Thymic Lobe A representative 3D image of GFP thymocytes within an intact thymic lobe. Image is rendered from one *z* stack at a single time point and is shown in a 360° rotation. Image size is 164 × 164 × 40 μm. Image was recorded approximately 140 μm below the thymic capsule. Corresponds to Figure 1.
(3.9 MB ZIP).

Video S2 Wild-Type GFP Thymocytes Migrating through an Intact Thymic Lobe Time-lapse image of dataset used to generate Video S1. Image is shown as a maximum projection of all *z* stacks. Corresponds to Figure 1. All movies were generated from 20 to 33 min of imaging and are played at six frames per second unless otherwise indicated.
(2 MB ZIP).

Video S3 4D Tracking of Wild-Type GFP Thymocytes Migrating through an Intact Thymic Lobe Same dataset as shown in Video S2 with tracks highlighted. Tracks were generated using 4D cell-tracking software. The fluorescence signal from GFP thymocytes is shown in green, and the positions of individual cells as determined by tracking software are represented as grey spheres. Tracks are color coded for time from blue (start of imaging) to light yellow (end of imaging).
(2.1 MB ZIP).

Video S4 4D Tracking of Wild-Type Thymocytes Reveals Distinct Migratory Behaviors A time-lapse image of GFP thymocytes in an intact thymic lobe with selected tracks highlighted. Image size is 104 × 104 × 40 μm. Note that the majority of thymocytes migrate slowly and turn frequently, as exemplified by the three MR^lo^ tracks on the right side. A small percentage of thymocytes migrate more rapidly and follow straight trajectories as exemplified by the MR^hi^ track highlighted on the left side.
(1.5 MB ZIP).

Video S5 MR^hi^ Cell Propulsion Is Associated with Polarized Morphology Time-lapse image of GFP thymocytes cropped to approximately 40 × 40 × 40 μm in the *x, y,* and *z* directions. Note the polarized morphology and dramatic shape changes of the MR^hi^ cell as it crawls from bottom to upper left corner. Video shown was generated from 5 min of imaging and is played at six frames per second. Corresponds to Figure 2C.
(418 KIB ZIP).

Video S6 P14 TCR Transgenic GFP Thymocytes in an Intact Thymic Lobe Time-lapse image of P14 TCR transgenic GFP thymocytes in an intact thymic lobe. The P14 TCR induces positive selection in this system. Note that a high proportion of thymocytes migrate rapidly and in straight trajectories compared to wild-type GFP thymocytes (Videos S2 and S3). Corresponds to Figure 4B.
(1.4 MB ZIP).

Video S7 P14 GFP Thymocyte Migration is Biased in the *z* Direction Time-lapse image of P14 TCR transgenic GFP thymocytes in intact thymic lobe is shown rotated to display the *x* and *z* dimensions. The same dataset was used to generate Video S6. Tracks of MR^hi^ cells are highlighted. Note that the majority of MR^hi^ tracks are oriented in the *z* direction.
(1.3 MB ZIP).

Video S8 5CC7 TCR Transgenic GFP Thymocytes in an Intact Thymic Lobe Time-lapse image of 5CC7 TCR transgenic GFP thymocytes in an intact thymic lobe. The 5CC7 TCR is nonselecting in this system. Note the almost complete absence of rapidly migrating thymocytes.
(1.1 MB ZIP).

## Abbreviations

CCR7: CC chemokine receptor 7
DN: double negative (CD4^–^CD8^–^)
DP: double-positive (CD4^+^ CD8^+^)
GFP: green fluorescent protein
MR: motility rate
MR^hi^: high motility rate
MR^lo^: low motility rate
SP: single-positive (CD4^+^CD8^–^ or CD4^–^CD8^+^)
TCR: T cell receptor
3D: three-dimensional
4D: four-dimensional
