# Correction: Directed Migration of Positively Selected Thymocytes Visualized in Real Time

**DOI:** 10.1371/journal.pbio.0030373

**Published:** 2005-10-11

**Authors:** Colleen M Witt, Subhadip Raychaudhuri, Brian Schaefer, Arup K Chakraborty, Ellen A Robey

In *PLoS Biology*, volume 3, issue 6: DOI: 10.1371/journal.pbio.0030160

The following statement in the last paragraph of the Results contains a factual error: “A total of 34% of cortical thymocytes expressing the P14 TCR had motility rates greater than 13 μm/min compared to approximately 2% for wild-type thymocytes expressing diverse TCRs.” The sentence should read as follows: “A total of 20% of cortical thymocytes expressing the P14 TCR had motility rates greater than 13 μm/min compared to approximately 2% for wild-type thymocytes expressing diverse TCRs.”

The authors apologize for any confusion this error may have caused.

